# Enabling precision rehabilitation interventions using wearable sensors and machine learning to track motor recovery

**DOI:** 10.1038/s41746-020-00328-w

**Published:** 2020-09-21

**Authors:** Catherine Adans-Dester, Nicolas Hankov, Anne O’Brien, Gloria Vergara-Diaz, Randie Black-Schaffer, Ross Zafonte, Jennifer Dy, Sunghoon I. Lee, Paolo Bonato

**Affiliations:** 1 Department of Physical Medicine & Rehabilitation, Harvard Medical School, Spaulding Rehabilitation Hospital, Boston, MA USA; 2 School of Health & Rehabilitation Sciences, MGH Institute of Health Professions, Boston, MA USA; 3 Department of Electrical and Computer Engineering, Northeastern University, Boston, MA USA; 4 College of Information and Computer Sciences, University of Massachusetts Amherst, Amherst, MA USA; 5 Wyss Institute for Biologically Inspired Engineering, Harvard University, Boston, MA USA

**Keywords:** Stroke, Brain injuries

## Abstract

The need to develop patient-specific interventions is apparent when one considers that clinical studies often report satisfactory motor gains only in a portion of participants. This observation provides the foundation for “precision rehabilitation”. Tracking and predicting outcomes defining the recovery trajectory is key in this context. Data collected using wearable sensors provide clinicians with the opportunity to do so with little burden on clinicians and patients. The approach proposed in this paper relies on machine learning-based algorithms to derive clinical score estimates from wearable sensor data collected during functional motor tasks. Sensor-based score estimates showed strong agreement with those generated by clinicians. Score estimates of upper-limb impairment severity and movement quality were marked by a coefficient of determination of 0.86 and 0.79, respectively. The application of the proposed approach to monitoring patients’ responsiveness to rehabilitation is expected to contribute to the development of patient-specific interventions, aiming to maximize motor gains.

## Introduction

By 2030, it is estimated that about 71 million Americans (973 million adults worldwide) will be 65 years of age or older^1^. The increase in life expectancy, due in part to recent advances in medical care, is unfortunately accompanied by an unprecedented and rapid increase in the prevalence of disability as older adults present with an accumulation of injuries and chronic conditions associated with disability^1,2^. Neurological conditions are common in older adults and affect as many as a billion people worldwide^3^. They are often associated with severe disability^4^ and cause significant societal burden^5^. Acquired brain injury (ABI), such as stroke and traumatic brain injury (TBI), has a high prevalence. About 2.4% of the US population experience lifelong disability due to stroke, and ~1.1% experience lifelong disability due to TBI^2,6,7^. Residual upper-limb motor deficits in these individuals contribute to loss of independence and poor quality of life.

Numerous studies have shown that rehabilitation interventions are beneficial across a number of neurological conditions as they result in a decrease in the severity of disability^8,9^. However, choosing the most effective intervention among the myriad of available rehabilitation approaches is challenging^10–12^. High variability in response to interventions aimed to restore upper-limb function is observed across patients^13–15^, hence pointing to the need for designing “precision rehabilitation” interventions that account for the unique characteristics of each individual. The need for developing patient-specific interventions is paramount in the broad field of medicine^16–18^ and is gradually emerging as a topic of great interest in the field of rehabilitation as investigators explore approaches relying on patients’ genotype^19–21^ and motor phenotype^22–24^ to develop subject-specific interventions.

In this context, it is important that rehabilitation specialists be provided with tools to monitor the motor recovery process, to assess if the ongoing intervention is leading to the anticipated clinical results, and to adjust the intervention if needed. Monitoring the motor recovery process includes assessing improvements in patients’ independence and participation in activities of daily living (ADL’s), which are important objectives of rehabilitation interventions as they are key factors to improve quality of life. Interventions are typically structured according to the International Classification of Functioning, Disability, and Health, which is referred to as the ICF model^25^. Rehabilitation specialists use this framework to evaluate interventions and rely on clinical outcome measures to capture different ICF domains (i.e., body function & structures, activity, and participation).

Clinical outcome measures are often based on the observation of subjects’ motor behaviors (e.g., to capture motor impairments and functional limitations). Unfortunately, these assessments are time-consuming and impractical to administer on a regular basis throughout the period of intervention. Outcome measures are too often collected only at baseline and at discharge. This is a problem because the lack of longitudinal data prevents rehabilitation specialists from examining the potential need for adjusting the intervention hence maximizing motor gains. To address this problem, researchers and clinicians have started to explore the use of wearable-sensing technology to collect the longitudinal data and derive estimates of clinical outcome measures (i.e., clinical scores).

Over the past decade, wearable technology has matured to the extent needed to provide an effective tool to monitor outcomes and facilitate delivering interventions^26–29^. Data can be collected in real-life conditions, thus enabling the assessment of upper-limb motor function where it counts the most, i.e., in the home and community setting^30,31^. This technology has tremendous potential for assessing the benefits of rehabilitation interventions^32^. Prior work by our research team has shown that accurate estimates of clinical scores capturing movement quality^33,34^ can be derived from accelerometer data collected during the performance of functional motor tasks. In addition, preliminary results suggest that a similar approach can be taken to assess the severity of upper-limb impairments^35,36^. However, obtaining accurate estimates of upper-limb impairments from wearable sensor data collected during the performance of functional tasks remains a challenge.

**Fig. 1:**
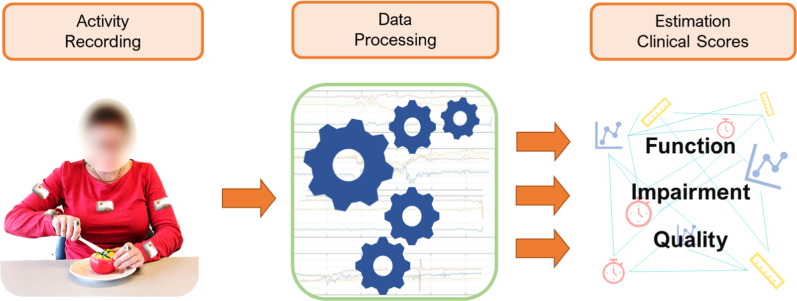
Schematic representation of the proposed approach. Data are collected using wearable sensors positioned on the upper limbs during the performance of functional tasks. Sensor data are fed to machine learning-based algorithms to derive estimates of clinical scores relevant to different ICF domains.

The methodology and results herein presented are an important step toward addressing the shortcomings of existing methods and demonstrate the feasibility of the approach schematically represented in Fig. 1. Data relevant to the assessment of upper-limb motor function are recorded in an unobtrusive manner during the performance of ADL’s using wearable sensors on the arm, forearm, and fingers. The data are then processed by relying on machine learning-based algorithms to estimate clinical scores across ICF domains (e.g., clinical scores capturing motor impairments, movement quality, and amount of use of the impaired limb). Accordingly, clinical outcomes are assessed throughout the intervention period using the data collected in the home and community settings.

## Results

### A novel approach to estimating clinical scores

The results herein presented show that wearable sensor data can be used to derive accurate estimates of clinical scores utilized in the clinic to capture the severity of motor impairments and the quality of upper-limb movement patterns. In the study, the upper-limb Fugl-Meyer assessment (FMA)^37^ scale was used to generate clinical scores of the severity of motor impairments, and the Functional Ability Scale (FAS)^38^ was used to generate clinical scores of the quality of movement. Wearable sensor data (i.e., accelerometer data) were collected during the performance of eight functional motor tasks taken from the Wolf Motor Function Test^38,39^ thus providing a sample of gross arm movements and fine motor control tasks. Machine learning-based algorithms were developed to derive accurate estimates of the FMA and FAS clinical scores from the sensor data.

### Study participants

A total of 37 study participants (16 stroke survivors and 21 TBI survivors) were recruited in the study. While the nature of the injury was different, all the subjects presented with residual upper-limb hemiparesis. The comparison between clinical characteristics of the stroke survivors and the TBI survivors did not show a statistically significant difference in clinical outcome measures (Table 1). The data also showed that the response to the intervention (i.e., baseline values vs. post-treatment values), as assessed using FMA and FAS scores, was similar in stroke and TBI survivors, although a large variability in motor gains was observed across study participants (Supplementary Fig. 1). Only the age significantly differed between the two populations, as expected because the average age at stroke onset is ~71 years for males and ~75 years for females^7^, whereas the incidence of TBI spans across the lifetime with a higher incidence in early childhood, late adolescence/early adulthood, and late life (i.e., among individuals 75 years of age and older)^40^. However, the age of the subjects is not expected to have any impact on the algorithms to derive FMA and FAS score estimates. Therefore, the datasets collected from these two populations were combined.

**Table 1:** Comparison of subjects’ clinical characteristics (stroke and TBI).

	Stroke (*n* = 16)	TBI (*n* = 21)	Total (*n* = 37)	Between groups^a^
Age, years, average ± SD	53.87 ± 25.74	34.03 ± 16.86	42.61 ± 18.98	*t*(35) = 3.65^‡^
Gender, male, *n* (%)	9 (56.3)	17 (81.0)	26 (70.3)	X^2^(1, 37) = 2.65
*Ethnicity*, n *(%)*				
Caucasian	13 (81.3)	20 (91.3)	33 (89.2)	X^2^(2, 37) = 2.86
African American	2 (12.5)	0 (0)	2 (5.4)	
Other	1 (6.3)	1 (4.8)	2 (5.4)	
Chronicity, months, average ± SD	13.03 ± 20.41	4.99 ± 8.17	8.46 ± 15.06	*t*(35) = 1.49
Hemiparesis, left, *n* (%)	11 (68.8)	6 (28.6)	17 (45.9)	X^2^(1, 37) = 5.90
Rehabilitation setting, inpatient, *n* (%)	10 (62.5)	20 (95.2)	30 (81.1)	X^2^(1, 37) = 6.35
Affected side, dominant, *n* (%)	6 (37.5)	13 (61.9)	19 (51.4)	X^2^(1, 37) = 2.17
Baseline FMA (/66), average ± SD	36.44 ± 10.43	37.10 ± 9.70	36.81 ± 9.89	*t*(35) = −0.20
Post FMA (/66), average ± SD	45.69 ± 12.92	51.67 ± 11.78	49.08 ± 12.48	*t*(35) = −1.47
Baseline total FAS (/5), average ± SD	2.98 ± 0.73	3.17 ± 0.73	3.08 ± 0.73	*t*(35) = −0.78
Post total FAS (/5), average ± SD	3.51 ± 0.82	3.90 ± 0.84	3.73 ± 0.84	*t*(35) = −1.39

*TBI* traumatic brain injury, *FMA-UE* Fugl-Meyer upper-extremity assessment, *FAS* Functional Ability Scale from the Wolf Motor Function Test, *SD* standard deviation, *TBI* traumatic brain injury.

^a^Chi-square tests were performed for categorical variables, and independent *t* tests were performed for continuous variables.

Significance level: ^‡^*P* < 0.01.

### Estimation of clinical scores using wearable sensor data

Prior work by our research team had shown that accurate FAS score estimates can be derived via the analysis of wearable sensor data^33,34,41^. In contrast, similar attempts to estimate FMA scores had led to unsatisfactory results^36^. Because previously proposed methods to derive FMA scores from wearable sensor data were shown to be inadequate and because of the high relevance of assessing impairments for planning and informing rehabilitation interventions for those affected by hemiplegia^37^, the work carried out in this study was primarily focused on achieving optimal estimates of FMA scores. The accuracy of the algorithms developed during the study was assessed by computing the root-mean-square error (RMSE) and the coefficient of determination (*r*^2^) of the clinical score estimates. The estimates’ bias was also derived to evaluate if the RMSE was primarily accounted for by the bias affecting the estimates, by the variability of the estimation error, or by a combination of the two. Inter-rater reliability data available for these clinical scales and the average change in clinical scores in response to the intervention were used to derive benchmarks for the accuracy of the clinical score estimates (see “Discussion”).

### Upper-limb impairment: estimation of FMA scores

The following four estimation algorithms were implemented and characterized. *Method 1* *(linear regression (*$\widehat {{\mathrm{FAS}}}$*))*: FMA scores were derived via linear regression of FAS estimates (i.e., $\widehat {{\mathrm{FAS}}}$) computed from the sensor data. *Method 2 (random forest)*: FMA scores were derived via analysis of sensor data, collected during the performance of eight functional motor tasks, using a random forest (RF) regression^42^ and subsequently aggregating the results obtained for all the motor tasks using a RF again. *Method 3 (balanced random forest)*: FMA scores were estimated by modifying Method 2 to balance the training set across FMA scores as part of the process by which data features were randomly selected to generate the decision trees of the RF. *Method 4 (proposed technique)*: FMA scores were estimated by modifying *Method 3* to add the $\widehat {{\mathrm{FAS}}}$ as input to the model.

Table 2 provides a summary of the results (i.e., RMSE and *r*^2^ values) obtained using these algorithms as well as the RMSE and *r*^2^ values of the FAS estimates derived from the wearable sensor data using a previously developed machine learning-based algorithm^34^.

**Table 2:** Accuracy of the FAS and FMA estimation algorithms investigated in the study.

	RMSE	*r*^*2*^
*FAS (0–5 points)*		
Random forest	0.38	0.79
*FMA (0–66 points)*		
Linear regression ($\widehat {\mathrm{{FAS}}}$)	7.79	0.47
Random forest	5.05	0.77
Balanced random forest	4.17	0.84
Proposed technique	3.99	0.86

Root-mean-square error (RMSE) and coefficient of determination (*r*^*2*^) values are shown for the FAS score estimates derived using a random forest-based algorithm as well as for the four methods implemented in the study to estimate FMA scores.

To implement the first of these methods (*linear regression* *(*$\widehat {\mathrm{{{FAS}}}}$*)*), a linear regression model was derived from the actual FAS and FMA scores (*r*^*2*^ = 0.75, Supplementary Fig. 2). Then the FAS scores estimated from the sensor data were fed to the linear regression model to derive FMA score estimates. The estimates were marked by a RMSE of 7.79 points and a *r*^*2*^ of 0.47 (Supplementary Fig. 3). Interestingly, the bias of the estimates was small (i.e., −0.10 points), thus showing that the RMSE was mostly accounted for by the variability of the estimation error. These results provided a benchmark for investigating methods to estimate FMA scores directly from wearable sensor data.

**Fig. 2:**
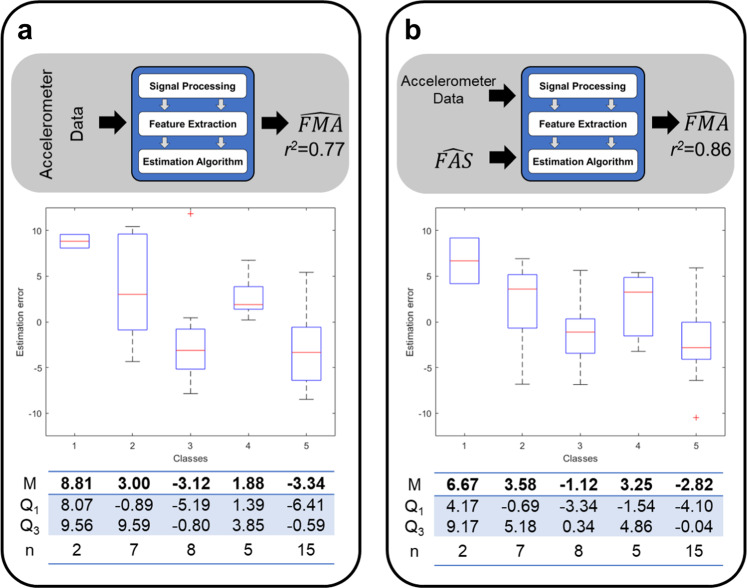
Estimation error distribution for two of the methods implemented in the study. **a** Random forest and **b** proposed technique. M median value, Q1 25th percentile, Q3 75th percentile, *n* number of subjects per class. See text for details.

The second of the above-mentioned methods (*random forest*) was implemented by using the cascade of two modules. The first module consisted of a set of algorithms to process individually the sensor data collected during the performance of each of the eight motor tasks utilized in the study. These algorithms derived data features from the accelerometer time series and fed them to a RF regression with 100 trees to generate FMA score estimates. The RMSE of the estimates derived from different motor tasks ranged from 6.17 to 10.77 points (Supplementary Table 1), with *r*^*2*^ = 0.65 as the highest coefficient of determination. It is worth pointing out that these results were obtained using the leave-one-subject-out cross-validation technique and that the highest coefficient of determination was obtained using data collected during a task combining gross arm movements and fine motor control tasks. A second module, implemented as a RF with 50 trees, was utilized to aggregate the estimates generated using data from the above-mentioned eight motor tasks. Using this second module led to a significant decrease in RMSE (i.e., RMSE = 5.05 points) and an increase in the coefficient of determination (*r*^*2*^ = 0.77). These results were obtained despite the fact that the distribution of clinical scores was nonuniform, as shown in Fig. 2a. The data shown in this figure was obtained by dividing the range spanned by the FMA scores of the study sample in five intervals (herein referred to as classes): FMA ≤ 30; 30 < FMA ≤ 38; 38 < FMA ≤ 47; 47 < FMA ≤ 56; and FMA > 56. Figure 2a shows both the uneven distribution of the datapoints (i.e., *n* = number of subjects per class ranging from 2 to 15) and the variability in the estimation error across classes.

To improve the accuracy of the FMA estimates, the algorithm was modified by balancing the classes during the training process as data features were selected to generate the trees of the RF. Herein, this method is referred to as *balanced random forest*. This approach led to a further improvement in the accuracy of the results. The RMSE decreased to 4.17 points, and the *r*^*2*^ increased to 0.84. The bias of the estimates remained low (i.e., −0.15 points).

**Fig. 3:**
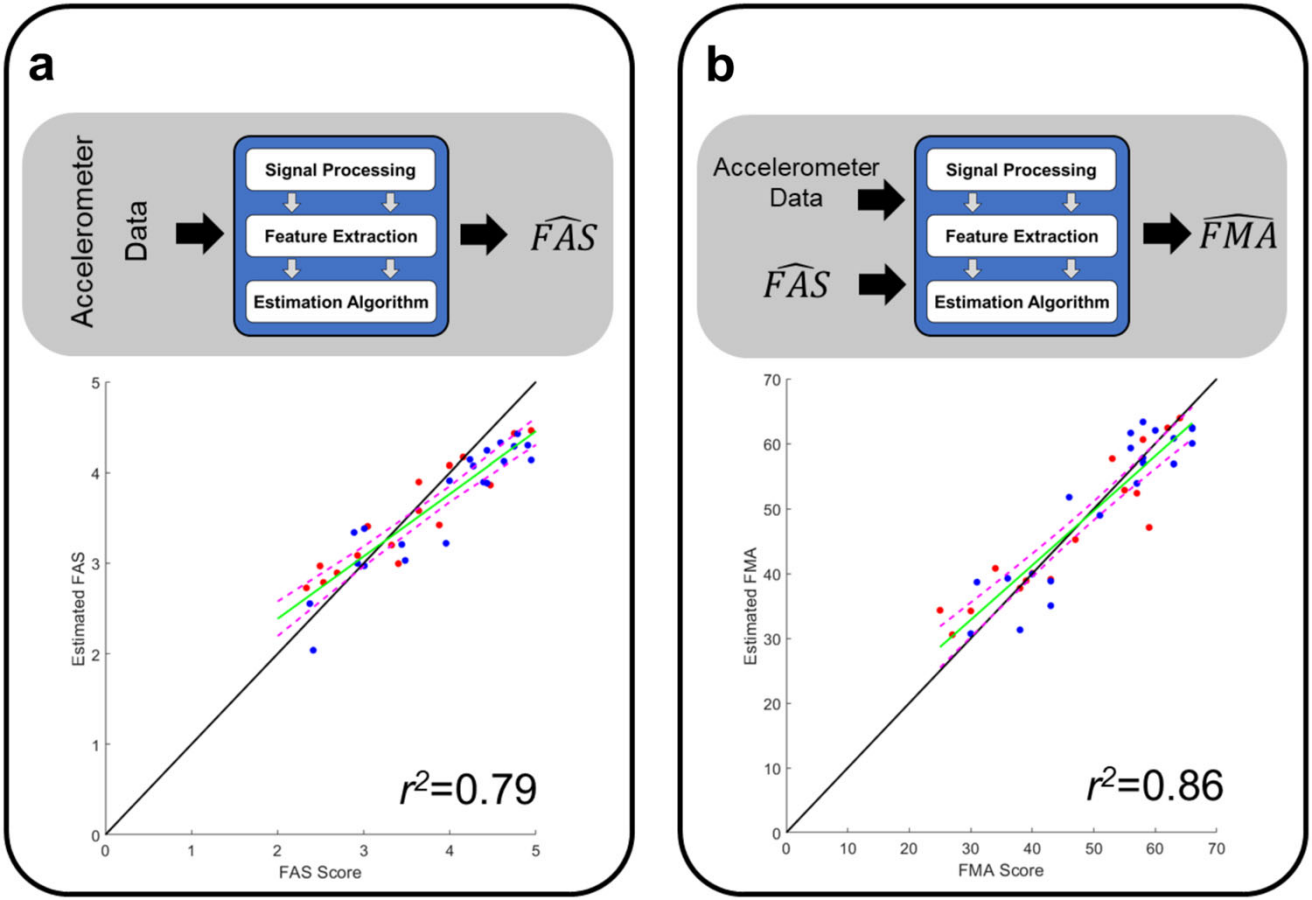
Estimation of movement quality (FAS) and motor impairment (FMA) clinical scores. **a** Results obtained using a technique previously developed by our research team. **b** Results obtained using the proposed technique (i.e., balanced RF with FAS estimates as an additional input). Blue and red circles represent data collected from TBI and stroke survivors. The green line is the linear regression of the estimates; the magenta dashed lines are the confidence intervals around the estimation. FAS Functional Ability Scale, FMA Fugl-Meyer assessment, RF random forest, TBI traumatic brain injury.

Finally, FAS score estimates (derived from the wearable sensor data, $\widehat {\mathrm{{FAS}}}$) were used as an additional input to the *balanced random forest* method. Herein, this algorithm is referred to as the *proposed technique*. The model performance further improved as the RMSE decreased to 3.99 points, and the coefficient of determination increased to 0.86. The bias remained substantially the same (i.e., −0.17 points). Figure 2b shows the impact of the algorithm on the error distribution across classes. The classes were set, as per Fig. 2a, by dividing the range spanned by the FMA scores in five intervals. Paired *t* tests (i.e., comparisons between the data shown in Fig. 2a for the *random forest* method and the data shown in Fig. 2b for the *proposed technique*) showed statistically significant improvements for the first, second, and fifth class. Overall, the results show a slight overestimation of the clinical scores for the most impaired subjects (FMA < 30 points) and a slight underestimation of the clinical scores for the least impaired subjects (FMA > 56 points). Figure 3b shows the FMA estimates obtained using this algorithm, which displayed a slight dependence of the estimates’ bias on the FMA value. Nonetheless, the sensor-based estimates matched well the actual FMA values. It is worth emphasizing that this algorithm represents a significant improvement over the benchmark data generated using the first of the algorithms implemented in the study (i.e., *linear regression* ($\widehat {{\mathrm{{FAS}}}}$) method), which led to estimates of the FMA scores marked by a *r*^*2*^ = 0.47. In contrast, the final implementation of the algorithm (i.e., the *proposed technique*) was marked by a *r*^*2*^ = 0.86.

### Movement quality: estimation of FAS scores

FAS estimates were derived using an algorithm, developed by our team in a previous study, that had been shown to have good performance^34^. When applied to the dataset of this study, the FAS estimation algorithm showed a RMSE of 0.38 points, a coefficient of determination of 0.79, and a bias equal to −0.15 points. Figure 3a shows the estimated FAS scores vs. the actual FAS scores for all the study participants.

The results shown in Fig. 3 are important because they demonstrate that both the quality of upper-limb movement patterns (Fig. 3a) and the severity of upper-limb motor impairments (Fig. 3b) can be accurately estimated by analyzing wearable sensor data using machine learning-based techniques.

## Discussion

The results of this study show that accurate estimates of the FAS and FMA scores can be derived by analyzing wearable sensor data, collected during the performance of functional motor tasks, using machine learning-based algorithms. FAS scores were estimated using a RF-based algorithm that we had previously developed and shown to be suitable to generate accurate estimates of FAS scores^34^. Analysis of the data collected in this study using this algorithm confirmed its suitability to generate accurate estimates of FAS scores (RMSE = 0.38 points, coefficient of determination *r*^2^ = 0.79). Prior work focused on estimating FMA scores, using an approach similar to the one used to derive FAS score estimates, had led to unsatisfactory results^36^. The algorithm resembled the first module of the above-described *random forest* method (i.e., *Method 2*), and it only provided acceptable results when the tenfold cross-validation technique was used. As it is known that this cross-validation technique is prone to overfitting, the leave-one-subject-out cross-validation technique was utilized in this study instead. This technique is known to be suitable to assess the generalizability of the proposed algorithms. As a case in point, the first module of the *random forest* method was implemented using the tenfold cross-validation technique as well as the leave-one-subject-out cross-validation technique. Supplementary Table 1 shows a comparison of the results obtained using these two cross-validation techniques, hence highlighting the need for developing a new approach. To address this problem, a new design of the machine learning-based algorithm to estimate FMA scores was proposed in this study. Estimates of the FMA scores generated using this algorithm were marked by a high coefficient of determination *r*^*2*^ = 0.86 and a RMSE = 3.99 points.

It is worth noticing that the proposed algorithm to estimate FMA scores performed well despite the relatively small sample size and the nonuniform distribution of the available clinical scores (i.e., an uneven number of datapoints across classes). This is because RF-based algorithms are robust to overfitting^42^ and hence particularly suitable when a small dataset is available. Furthermore, we chose to rebalance the training set as part of the process by which datapoints were randomly selected to build the decision trees of the RF. This technique led to a decrease in the level of association between the variability in the estimation error and the actual FMA score. With a larger dataset, one would expect to achieve a “near-uniform” distribution of the estimation error. That would further reduce the estimation bias. Then highly accurate estimates of the FMA scores could be generated by averaging estimates derived from repeated measures of motor task performance. This approach would allow clinicians to reduce the variance of the FMA estimates. The same approach could be taken to generate highly accurate FAS score estimates. Using this technique, FMA and FAS score estimates obtained via analysis of the sensor data could match the high reliability of the FMA and FAS clinical scales. Both the FMA and the FAS clinical scales have been shown to be marked by high intraclass correlation coefficients (ICC) (= 0.96 for the FMA^43,44^ and = 0.88 for the FAS^39^). These ICC values could be related to the number of repeated measures needed to reduce the FMA and FAS estimation errors when deriving the clinical scores from sensor data. Similarly, one could derive the number of repeated measures needed to make the estimation errors negligible compared to the changes in clinical scores observed in response to the clinical intervention of interest.

The feasibility of the above-described approach would rely on deriving clinical score estimates using wearable sensor data, which could be collected with virtually no patients’ and clinicians’ burden. Besides, we designed the proposed technique to be suitable to derive clinical score estimates from data collected during the performance of functional motor tasks. As functional motor tasks are part of the performance of ADL’s, FAS and FMA score estimates could then be derived via the analysis of data collected in the home and community setting. Future work should fully enable this approach by further improving the unobtrusiveness and ease of use of wearable sensors and by developing fully automated data analysis procedures, for instance, for the segmentation of the sensor data based on detecting data characteristics associated with the performance of motor tasks suitable to derive reliable estimates of clinical scores.

**Fig. 4:**
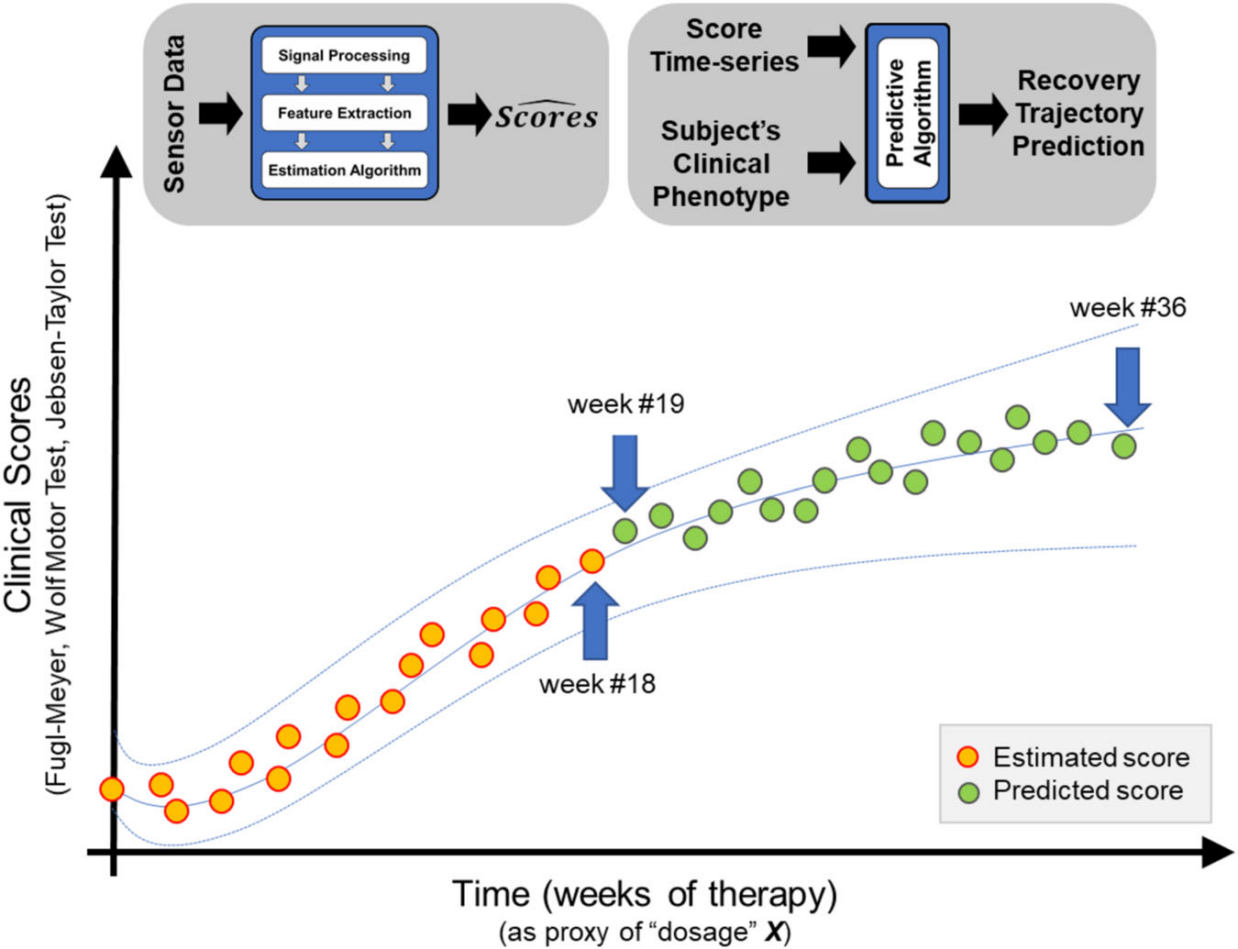
Monitoring the motor recovery trajectory using wearable sensors. The time series represent the recovery trajectory of a hypothetical subject undergoing rehabilitation. Clinical scores estimated via the analysis of wearable sensor data (orange circles) provide measures of arm ability across domains of the ICF. Predicted scores (green circles) derived using a model based on the time series of estimated clinical scores (orange circles) and subject’s clinical phenotype provide estimates of the anticipated response to the intervention. A fitting function (e.g., a polynomial equation) represents the recovery trajectory. Confidence intervals are provided for the estimated and predicted scores.

These methods would allow clinicians to track the motor recovery trajectory of stroke and TBI survivors as schematically represented in Fig. 4. The figure shows a hypothetical case in which a subject undergoes a 36-week intervention. During this period of time, wearable sensors are used to monitor the subject. After 18 weeks, clinical score estimates, derived from the sensor data, are available and define the motor recovery trajectory observed in response to the intervention until that point in time (orange circles in Fig. 4). The data can be used by rehabilitation specialists to assess if the patient is responding adequately to the ongoing intervention or if an adjustment to the intervention strategy is needed. Importantly, the clinical score time series can be used to predict the patient’s response to the intervention for the remaining 18 weeks, namely from week 19th to week 36th of the intervention period (green circles in Fig. 4). This can be achieved by using, for instance, Gaussian Process Regression models^45^. Such models could account for the patient’s clinical phenotype and hence generate predictions based on both the clinical score time series and the anticipated response to the intervention based on the patient’s clinical characteristics.

Many models have been proposed to predict clinical scores at discharge using clinical data collected at baseline^46–48^. For instance, corticospinal tract integrity observed via imaging techniques shortly after a stroke has been associated with an expected 70% proportional restitution of motor impairments at 3 months^49–51^. This value could be utilized as an “intervention target” and the above-described algorithms, to generate predictions of the motor recovery trajectory, could be utilized to assess whether a given patient is “on track” to achieve the expected level of recovery. In contrast, when the corticospinal tract is compromised, rehabilitation specialists would expect that patients would typically display a modest level of recovery. However, researchers are exploring pharmacotherapies and regenerative medicine approaches that have the potential for achieving a significant impact on the recovery trajectory and, importantly, the amount of restitution. In this context, the above-described algorithms could be relied upon to assess and predict the effectiveness of a given therapeutic intervention.

The approach described in this hypothetical clinical scenario captures the essence of precision rehabilitation in which clinicians design patient-specific interventions, set clinical objectives, track patient’s response using wearable sensors, and periodically evaluate the effectiveness of the ongoing intervention based on the recovery trajectory defined by the time series of clinical score estimates derived from wearable sensor data. The ability of tracking and predicting clinical scores is key in this context and, we argue, will enable patient-specific interventions marked by unprecedented motor gains.

## Methods

### Study participants

Subjects were prospectively recruited for a longitudinal study. Inclusion criteria were: (1) unilateral stroke (hemorrhagic or embolic) or focal traumatic brain injury; (2) age ranging from 18 to 80 years at enrollment; (3) currently undergoing upper-limb rehabilitation (inpatient or outpatient); and (4) severe-to-moderate upper-limb impairment as determined by a score between 15 and 55 points out of 66 on the upper-limb FMA scale. Patients with a Mini-Mental State Examination test^52^ score below 24 and not able to follow a three-step command were excluded from the study. Study procedures were reviewed and approved by the Spaulding Rehabilitation Hospital Institutional Review Board (IRB). Written informed consent was obtained from each study participant or a legally authorized representative. All study procedures were carried out in accordance with relevant guidelines and regulations.

### Experimental procedures

Enrolled subjects participated in two identically structured visits (described below), the first at baseline and the second at discharge. During the study visits, a research therapist administered a battery of standardized clinical tests, including the upper-limb portion of the FMA and the FAS.

The upper-limb FMA is a clinical test designed to evaluate motor impairments that has been tested extensively in the stroke population^37^. A total of 33 items assessing voluntary movement, reflexes, grasp, and coordination are tested; each item is rated on a three-point ordinal scale. A score of 0 is assigned when the subject cannot perform the item; a score of 1 is assigned when the subject can only partially perform the item; and a score of 2 is assigned when the subject performs the item flawlessly. The total score is the sum of the scores for each item of the scale. The maximum achievable score is 66 points. The scale has excellent inter-rater and intra-rater reliability as well as excellent construct validity^53^.

The FAS is used to assess the quality of movement via observation of the performance of the items of the Wolf Motor Function Test (WMFT). The WMFT is commonly used to quantify upper-limb motor function with timed functional tasks^38^. It consists of 17 items progressing from proximal to distal and from least to most complex upper-limb movements. Each item is used to assess speed and movement quality. Two items are meant to assess the strength and are scored separately from the rest of the scale. The FAS relies on a six-point ordinal scale, where a score of 0 is assigned when the task is not attempted, and a score of 5 is assigned to movements that appear to be “normal”. Scores from 1 to 4 are associated with the presence and severity of compensatory movements, the use of the unaffected upper-limb to support the affected upper-limb, the speed and smoothness of movement during the performance of the task relative to normative. Higher FAS scores indicate better quality of movement. The clinimetric properties of the clinical scale are excellent^39^. The test was performed in a standardized manner, according to guidelines developed by Taub et al.^54^.

**Fig. 5:**
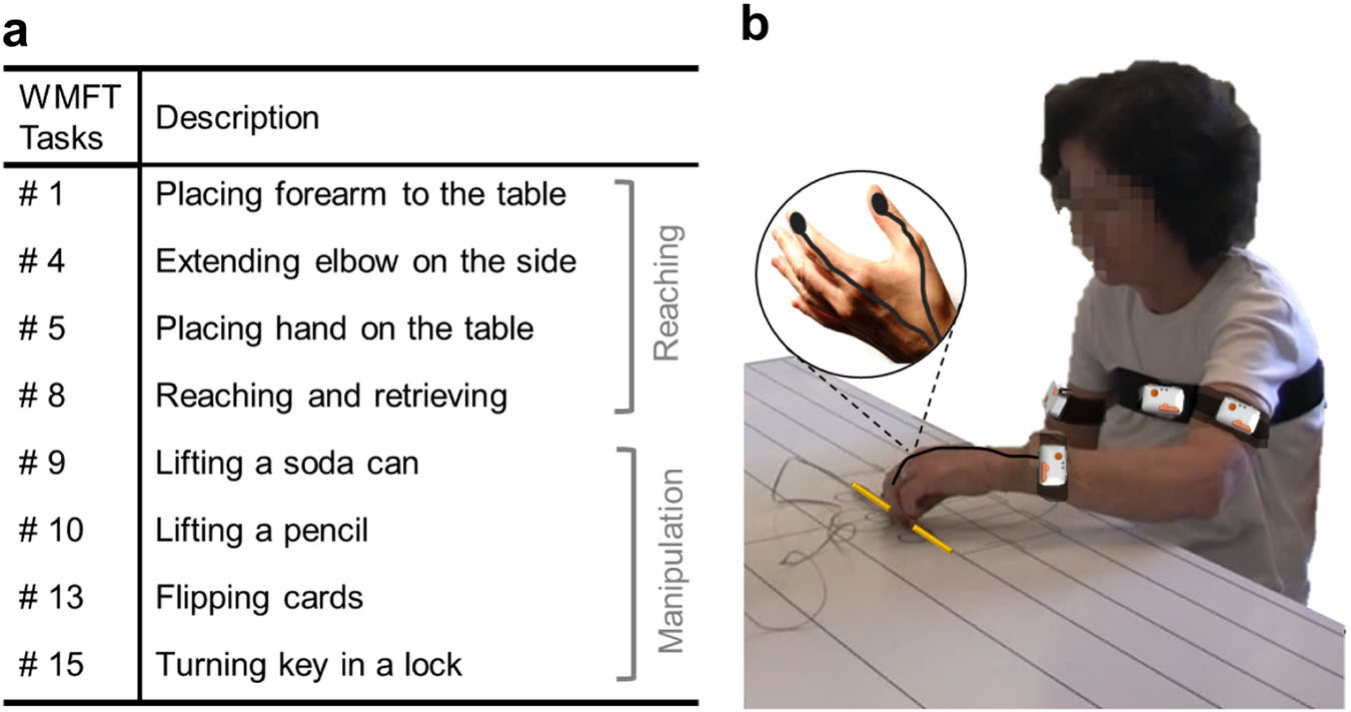
Experimental procedures. Functional tasks performed during the data collections **a** and position of the wearable sensors **b**.

After the clinical tests, a total of six wearable sensors (Shimmer2 by Shimmer Sensing, Dublin, Ireland) were placed on the chest (sternum height), arm (mid-biceps, frontal), and wrist (above radius and cubitus styloid, dorsal) bilaterally, and on the index and thumb (dorsal part of the distal phalange) of the affected upper limb using self-adherent wrap (Coban, 3 M) (Fig. 5b). All the units were equipped with three-axis accelerometers, except for the units positioned on the thumb and index finger that were equipped with two-axis accelerometers. All the units were synchronized and programmed using a dedicated software platform.

Subjects were then instructed to perform selected motor tasks from the WMFT. Eight WMFT motor tasks were selected and performed by study participants (Fig. 5a). The tasks selected for the study were previously reported to be suitable to estimate FAS scores^34^ and provided encouraging results when utilized to estimate FMA scores^36^. Each task was timed, and its performance quality evaluated using FAS-based criteria. Four tasks evaluated arm-reaching capacity: a forearm to table-side (WMFT-1), extend elbow-side (WMFT-3), hand to the table (WMFT-5), and reach and retrieve (WMFT-8). The remaining four tasks assessed manipulation ability: lift a can of soda (WMFT-9), lift a pencil (WMFT-10), flip cards (WMFT-13), and turn key in a lock (WMFT-15). In order to account for possible within-subject variability, each task was performed up to three times, and each occurrence was scored separately. Both sessions were video-recorded for off-line review by research therapists.

### Data analysis

Potential differences between stroke and TBI study participants’ clinical characteristics were assessed using Chi-Square tests for categorical variables and independent *t* tests for continuous variables. Statistical tests were performed using SPSS (Statistical Packages for Social Sciences, version 23.0; SPSS Inc., Chicago, IL, USA). Data were checked for normality of distribution, significance was set at α = 0.05, and *P* values were adjusted for multiple comparisons using a Holm correction^55^.

Average FAS scores were derived from the scores assigned to each trial for a given item of the scale. Average scores for the eight tasks utilized in the study were used in the equation below to derive the FAS total score^34^. The resultant FAS score ranged between 0 and 5 points.

$${\mathrm{{FAS}}}_{{\mathrm{Total}}} = \frac{{\left( {{\sum} {{\mathrm{FAS}}_i \times 1.78} } \right) + 2.97}}{{15}}$$

### Sensor data processing

The raw accelerometer data was imported in the MATLAB programming environment (The MathWorks Inc, Natick, MA, USA), and custom scripts were used to implement the processing steps described below.

First, the accelerometer data recorded during the performance of the WMFT tasks were segmented in order to select the time intervals during which each motor task was performed. The segmentation was accomplished using a digital marker recorded during the data collection to identify when subjects started and completed the performance of each motor task.

Then, data features were extracted and selected. To that aim, accelerometer time series were low-pass filtered with a cut-off frequency of 8 Hz (sixth-order Butterworth filter) to remove high-frequency noise and then high-pass filtered with a cut-off frequency of 0.25 Hz (sixth-order Butterworth filter) to isolate the acceleration components of movements and minimize the effects of postural adjustments. The magnitude time series for displacement, velocity, acceleration, and jerk were derived by combining the data for the three axes (or two axes when the unit was equipped with two axes) of each sensor unit. Based on prior work that had shown their suitability to derive FAS scores^33,34,36^, the following data features were extracted from the sensor time series: (1) minimum, maximum, and mean values, (2) root- mean-square value, (3) ratio of the magnitude of the dominant frequency and total signal energy, (4) jerk, (5) skewness, (6) signal entropy, (7) kurtosis, (8) correlation coefficients derived from the time series computed for different axes, and (9) duration of the data segments associated with the performance of each movement component. Correlation coefficients derived from the magnitude time series of the accelerometer data were computed for all the sensors (i.e., sensors positioned on the fingers, wrist, arm, and sternum). These correlation coefficients were meant to capture the characteristics of relative movements of different body segments, including compensatory movements such as leaning forward during the performance of an arm-reaching movement. The selection of data features was achieved using a correlation-based algorithm^56^.

To estimate the FAS clinical score from the sensor data, a RF regression^42^ was used to estimate the FAS scores for each repetition of the motor tasks based on data features that were computed and selected, as explained above. The number of RF trees was set to 100. A RF-based approach was chosen because of its robustness when processing datasets of small size. The RF-based algorithm was implemented using the leave-one-subject-out cross-validation method to avoid overfitting.

**Fig. 6:**
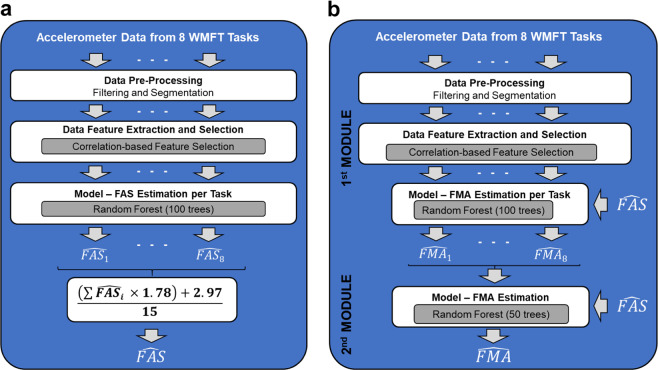
Graphical illustration of the data analysis pipeline. **a** FAS score estimation pipeline; and **b** FMA score estimation pipeline. FAS Functional Ability Scale, FMA Fugl-Meyer assessment.

For each subject, the estimated FAS scores for all the repetitions of a given motor task were averaged. Then, the average FAS scores per task were added across tasks. The resultant score was used as input to a linear equation to estimate the total FAS score. The equation utilized to aggregate FAS estimates, computed using data for individual tasks, was derived in earlier work^34^. Figure 6a shows a schematic representation of the full data analysis pipeline to estimate the FAS scores from the wearable sensor (i.e., accelerometer) data.

To estimate the FMA clinical score from the sensor data, the following four different methods were implemented to generate FMA score estimates.

#### Method 1 (linear regression ($\widehat {{\mathrm{FAS}}}$))

FAS estimates (i.e., $\widehat {{\mathrm{FAS}}}$) were first computed using an algorithm previously developed by our research group to derive FAS estimates from sensor data^34^. Then, FMA scores were derived via a linear regression model of the FAS estimates (i.e., $\widehat {{\mathrm{FAS}}}$). The linear regression model was derived using the actual FMA and FAS scores (i.e., those generated by rehabilitation specialists).

#### Method 2 (random forest)

A RF regression with 100 trees was used to generate FMA score estimates for each of the eight functional motor tasks utilized in the study. Data features were derived and selected based on the algorithms described above. Then, the FMA score estimates for each motor task were combined using a RF with 50 trees. The output of this RF provided an estimate of the FMA total score.

#### Method 3 (balanced random forest)

This method was derived by modifying Method 2. Specifically, the RFs utilized to generate the FMA score estimates for each motor task were trained using balanced datasets as part of the process by which data features are randomly selected to generate the decision trees of the RF. The balancing of the training set was obtained by using five classes: (1) FMA ≤ 30; (2) 30<FMA ≤ 38; (3) 38<FMA ≤ 47; (4) 47<FMA ≤ 56; and (5) FMA > 56.

#### Method 4 (proposed technique)

This method was derived by modifying Method 3. Specifically, an input was added to each RF. The additional input was used to feed the algorithm with estimates of the FAS scores (i.e., $\widehat {{\mathrm{FAS}}}$). Figure 6b shows a schematic representation of the final data analysis pipeline to estimate the FMA scores using wearable sensor data.

### Reporting summary

Further information on research design is available in the Nature Research Reporting Summary linked to this article.

## Supplementary information

Supplementary Information

Reporting Summary

## Data Availability

The data collected and analyzed in the study herein presented are available upon request to be sent to the corresponding author.

## Code availability

The code utilized in the study was developed in the MATLAB programming environment (The MathWorks Inc, Natick MA, USA), version R2019b. The code is available upon request to be sent to the corresponding author.
