# Supplementary material for: Enabling precision rehabilitation interventions using wearable sensors and machine learning to track motor recovery
Source: NPJ Digit Med. 2020 Sep 21;3:121. doi: 10.1038/s41746-020-00328-w (PMC7506010; doi:10.1038/s41746-020-00328-w)
Supplement: Supplementary file 1 — Supplementary Information [file 41746_2020_328_MOESM1_ESM.pdf]

Supplementary Materials

Supplementary Figure 1	Variability across study participants in their response to rehabilitation interventions.
Supplementary Figure 2	Relationship between upper-limb impairments (i.e., FMA scores) and quality of movement (i.e., FAS scores).
Supplementary Figure 3	Estimation of impairment severity from quality of movement estimates.
Supplementary Table 1	Comparison of the proposed method with prior work (Del Din et al., 2011) attempting to generate FMA estimates on a task-by-task basis

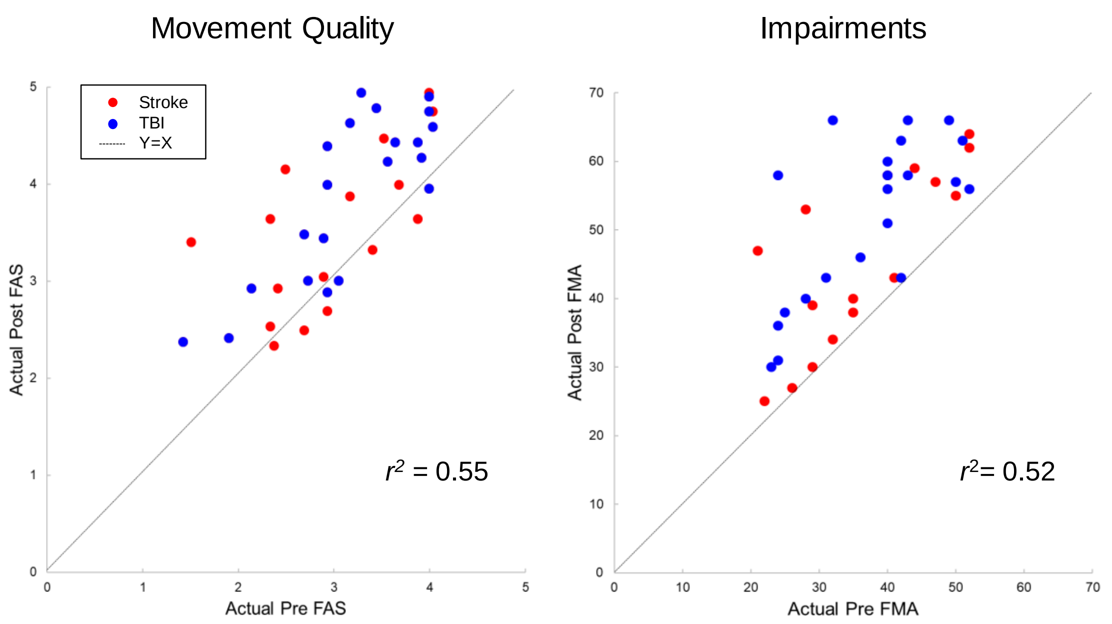

Supplementary Figure 1. **Variability across study participants in their response to rehabilitation interventions**. Left panel: movement quality as assessed using the FAS. Right panel: severity of motor impairments as assessed using the FMA. r^2: coefficients of determination between pre- and post-intervention scores ($p<0.001$ for both outcomes). The change in clinical score was $\Delta = 0.65\pm0.58$ (mean ± standard deviation) for the FAS and $\Delta = 12.27\pm8.67$ for the FMA.

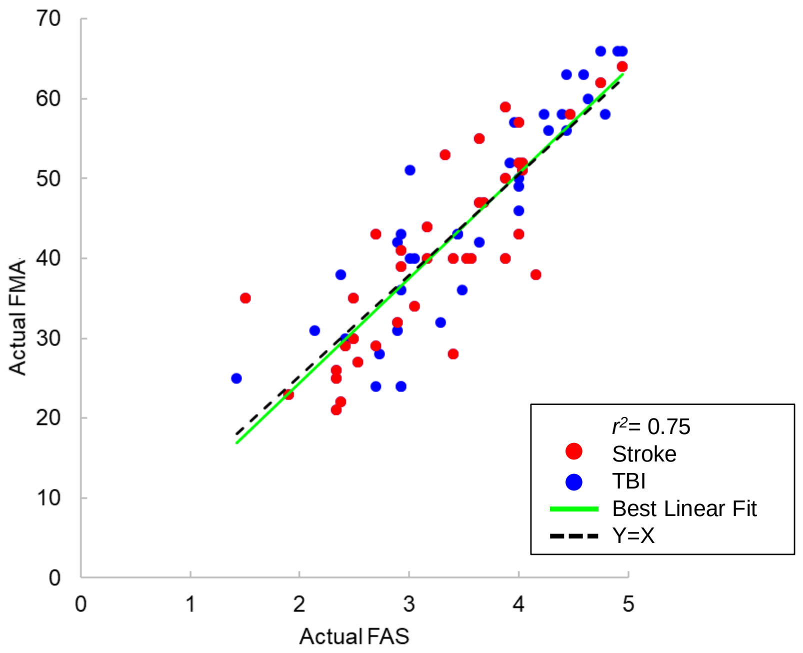

***Supplementary Figure 2.* Relationship between upper-limb impairments (i.e., FMA scores) and quality of movement (i.e., FAS scores).** The plot shows, as somewhat expected, a correlation between impairments and quality of movement.

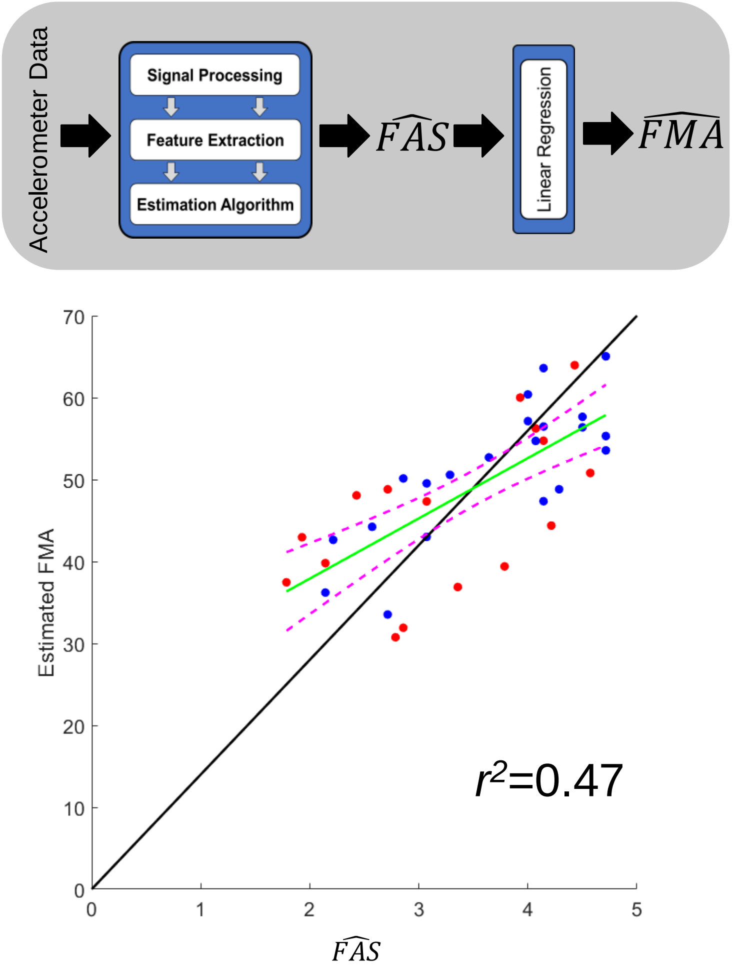

Supplementary Figure 3. **Estimation of impairment severity from quality of movement estimates.** FMA estimation with a linear regression using estimated FAS scores. FAS scores were previously estimated using a Random Forest algorithm based on wearable sensor (i.e., accelerometer) data. Blue and red circles represent datapoints for TBI and stroke survivors, respectively. The green line is the linear regression of the datapoints. The magenta dashed lines represent the confidence intervals.

		Task 1	Task 4	Task 5	Task 8	Task 9	Task 10	Task 13	Task 15
		Forearm table (side)	*Extend elbow (weight)*	*Hand to table*	*Reach and retrieve*	*Lift can*	*Lift pencil*	*Flip cards*	*Turn Key*
RF 100 10-fold	RMSE	7.39	6.99	6.71	7.33	5.66	6.81	6.41	5.36
	r^2	*0.37*	*0.51*	*0.52*	*0.45*	*0.68*	*0.53*	*0.57*	*0.71*
RF 100 LOO	RMSE	10.77	8.67	8.64	8.75	6.83	8.17	7.35	6.17
	r^2	*0.01*	*0.33*	*0.36*	*0.30*	*0.60*	*0.38*	*0.49*	*0.65*

***Supplementary Table 1.* Comparison of the proposed method with prior work (Del Din et al., 2011) attempting to generate FMA estimates on a task-by-task basis.** RF 100: Random Forest 100 trees; 10-fold: 10-fold cross-validation; LOO: Leave-One-subject-Out cross-validation; RMSE: root mean square error; r^2: coefficient of determination
