# Supplementary material for: Enabling precision rehabilitation interventions using wearable sensors and machine learning to track motor recovery
Source: NPJ Digit Med. 2020 Sep 21;3:121. doi: 10.1038/s41746-020-00328-w (PMC7506010; doi:10.1038/s41746-020-00328-w)
Supplement: Supplementary file 2 — Reporting Summary [file 41746_2020_328_MOESM2_ESM.pdf]

nature research

Corresponding author(s): Paolo Bonato, PhD

Last updated by author(s): 08/05/2020

Reporting Summary

Nature Research wishes to improve the reproducibility of the work that we publish. This form provides structure for consistency and transparency in reporting. For further information on Nature Research policies, see our Editorial Policies and the Editorial Policy Checklist.

Statistics

For all statistical analyses, confirm that the following items are present in the figure legend, table legend, main text, or Methods section.

n/a	Confirmed	
☐	☒	The exact sample size (*n*) for each experimental group/condition, given as a discrete number and unit of measurement
☐	☒	A statement on whether measurements were taken from distinct samples or whether the same sample was measured repeatedly
☐	☒	The statistical test(s) used AND whether they are one- or two-sided<br>*Only common tests should be described solely by name; describe more complex techniques in the Methods section.*
☒	☐	A description of all covariates tested
☐	☒	A description of any assumptions or corrections, such as tests of normality and adjustment for multiple comparisons
☐	☒	A full description of the statistical parameters including central tendency (e.g. means) or other basic estimates (e.g. regression coefficient) AND variation (e.g. standard deviation) or associated estimates of uncertainty (e.g. confidence intervals)
☐	☒	For null hypothesis testing, the test statistic (e.g. *F*, *t*, *r*) with confidence intervals, effect sizes, degrees of freedom and *P* value noted<br>*Give P values as exact values whenever suitable.*
☒	☐	For Bayesian analysis, information on the choice of priors and Markov chain Monte Carlo settings
☒	☐	For hierarchical and complex designs, identification of the appropriate level for tests and full reporting of outcomes
☒	☐	Estimates of effect sizes (e.g. Cohen's *d*, Pearson's *r*), indicating how they were calculated

Our web collection on statistics for biologists contains articles on many of the points above.

Software and code

Policy information about availability of computer code

Data collection	We used the software tools made available by the manufacturer of the sensor units utilized in the study (Shimmer Research, Dublin, Ireland) and LabView virtual instrument modules that we utilized to synchronize the sensor units.
Data analysis	We used custom MATLAB scripts (The MathWorks Inc, Natick, MA, USA) to implement the algorithms described in the manuscript. We used SPSS (Statistical Packages for Social Sciences, version 23.0; SPSS Inc., Chicago, IL, USA) to perform the reported statistical analyses.

For manuscripts utilizing custom algorithms or software that are central to the research but not yet described in published literature, software must be made available to editors and reviewers. We strongly encourage code deposition in a community repository (e.g. GitHub). See the Nature Research guidelines for submitting code & software for further information.

Data

Policy information about availability of data

All manuscripts must include a data availability statement. This statement should provide the following information, where applicable:

- Accession codes, unique identifiers, or web links for publicly available datasets
- A list of figures that have associated raw data
- A description of any restrictions on data availability

The data collected in the study and the code developed by our research team during the study are available upon request to be sent to the corresponding author.

Field-specific reporting

Please select the one below that is the best fit for your research. If you are not sure, read the appropriate sections before making your selection.

☒ Life sciences ☐ Behavioural & social sciences ☐ Ecological, evolutionary & environmental sciences

For a reference copy of the document with all sections, see nature.com/documents/nr-reporting-summary-flat.pdf

Life sciences study design

All studies must disclose on these points even when the disclosure is negative.

Sample size	The adequacy of the sample size was assessed based on demonstrating the generalizability of the proposed machine learning-based algorithms using the leave-one-subject-out cross-validation technique. This is considered the gold standard in the field of machine learning. Besides, for the correlation analyses performed to assess the agreement between the sensor-based estimates of the clinical scores and the scores generated by clinicians we showed a power exceeding 90%.
Data exclusions	Subjects lost to follow-up thus resulting in incomplete datasets were excluded.
Replication	We are confident that the results reported in the manuscript could be easily replicated because 1) we used a cross-validation technique that is considered the gold standard in machine learning to demonstrate generalizability of the proposed methods, and 2) the results obtained in the study, although they represent a substantial improvement compared to previous projects, are consistent in many regards with previous preliminary results.
Randomization	Subjects were not randomized because our data collection was limited to assessing arm impairments and quality of movements longitudinally. No intervention was provided as part of the study procedures.
Blinding	Participants were not allocated to an intervention for the purpose of the study. Therefore, no blinding of investigators was needed.

Reporting for specific materials, systems and methods

We require information from authors about some types of materials, experimental systems and methods used in many studies. Here, indicate whether each material, system or method listed is relevant to your study. If you are not sure if a list item applies to your research, read the appropriate section before selecting a response.

Materials & experimental systems

n/a	Involved in the study	
☒	☐	Antibodies
☒	☐	Eukaryotic cell lines
☒	☐	Palaeontology and archaeology
☒	☐	Animals and other organisms
☐	☒	Human research participants
☒	☐	Clinical data
☒	☐	Dual use research of concern

Methods

n/a	Involved in the study	
☒	☐	ChIP-seq
☒	☐	Flow cytometry
☒	☐	MRI-based neuroimaging

Human research participants

Policy information about studies involving human research participants

Population characteristics	16 stroke and 21 traumatic brain injury (TBI) survivors were recruited, all presenting with hemiparesis due to their brain injury. 70% of the participants were male, which was expected as TBI is affecting primary males. An effort was made to include participants from all ethnic backgrounds. However, the majority of the study participants described themselves as Caucasian. Lastly, the age range varied with TBI survivors being on average ~20 years younger than stroke survivors.
Recruitment	Study participants were recruited via the inpatient and outpatient clinics at Spaulding Rehabilitation Hospital. Participation was voluntary. No clinician involved in the patients' care was involved in the recruitment and/or data collection procedures for this research study.
Ethics oversight	Spaulding Rehabilitation Hospital Institutional Review Board (IRB).

Note that full information on the approval of the study protocol must also be provided in the manuscript.
